# Luteolin Alleviates Vascular Senescence Through Retinoic Acid–Peroxisome Proliferator-Activated Receptor Signaling and Lipid Metabolism Remodeling Combined with Multi-Omics Analysis

**DOI:** 10.3390/nu17223607

**Published:** 2025-11-19

**Authors:** Huasong Bai, Hongchen Jin, Tong Liu, Yulong Yin, Hengyan Wang, Siyu Ruan, Yunliang Li, Zhanzhong Wang

**Affiliations:** 1 Nourse Science Centre for Pet Nutrition, Wuhu 241200, China; baihuasong_cx@163.com (H.B.); jinhongchen_cx@163.com (H.J.); liutong_cx@163.com (T.L.); wanghengyan_cx@163.com (H.W.); 2 Institute of Subtropical Agriculture, Chinese Academy of Sciences, Changsha 410125, China; yinyulong@isa.ac.cn; 3 College of Tea and Food Science Technology, Jiangsu Vocational College of Agriculture and Forestry, 19 Wenchangdong Road, Jurong, Zhenjiang 212400, China; siyuruan@126.com; 4 School of Food and Biological Engineering, Jiangsu University, 301 Xuefu Road, Zhenjiang 212013, China

**Keywords:** dietary intervention, luteolin, peroxisome proliferator-activated receptor, retinoic acid, transcriptomics, vascular aging

## Abstract

**Background:** Although luteolin (Lut) is well recognized for its anti-inflammatory and antioxidant effects, its potential role in preventing vascular senescence remains underexplored in primary vascular aging. This study aimed to investigate the anti-vascular-aging effects of Lut in both cellular and murine aging models and to elucidate its conserved molecular mechanisms across species. **Methods:** Canine and feline vascular endothelial cells (cVECs and fVECs) were subjected to doxorubicin-induced senescence, while senescence-accelerated mice prone 8 (SAMP8) received an 8-week dietary supplementation with Lut. Senescence markers, inflammatory cytokines, antioxidant activities, vascular biomechanics, and histological changes were assessed. Transcriptomic and metabolomic analyses were combined to identify molecular pathways. Statistical significance was determined by one-way analysis of variance with Tukey’s or Games–Howell post hoc tests (*p* < 0.05). **Results:** Lut markedly reduced senescence-associated β-galactosidase activity, suppressed interleukin-6 and matrix metalloproteinase expression (*p* < 0.05), and enhanced superoxide dismutase activity and nicotinamide adenine dinucleotide levels (*p* < 0.05) in cVECs, fVECs, and SAMP8 sera. In aged mice, Lut alleviated arterial wall thickening and vascular inflammation, improved vascular biomechanics and systemic oxygenation (*p* < 0.05), and attenuated cardiac and hepatic inflammatory infiltration. Multi-omics analyses in cVECs revealed that Lut targets aldehyde dehydrogenase 1 to increase 9-cis retinoic acid, thereby activating the retinol X receptor–peroxisome proliferator-activated receptor (PPAR) network, which accelerates lipid clearance and oxidation. Consistent activation of this pathway was validated in murine vascular transcriptomes. **Conclusions:** These findings demonstrate that Lut delays vascular aging by activating the retinoic acid–PPAR axis and reprogramming lipid metabolism. This conserved mechanism was consistently observed in doxorubicin-induced cVEC senescence and the SAMP8 model, underscoring the robustness of Lut’s action across distinct contexts of vascular aging.

## 1. Introduction

With advancing age, organisms experience a progressive disruption of homeostatic regulation, accompanied by functional decline across multiple organs. This deterioration is driven by various cellular events, including cell-cycle arrest, mitochondrial and metabolic dysfunction [1], chronic low-grade inflammation [2], and extracellular matrix remodeling [1,2,3]. Among organ systems, the vasculature is most susceptible to aging, which is manifested as endothelial dysfunction [4,5,6], reduced nitric oxide bioavailability [7,8], heightened oxidative and inflammatory signaling [9,10], increased arterial stiffness, and structural remodeling of the vessel wall [4,5,6,7,8,9,10,11,12,13,14]. Senescence of cells activates the senescence-associated secretory phenotype (SASP), which disrupts vascular homeostasis. These processes drive the progression of atherosclerosis, hypertension, cerebrovascular disease, and pulmonary vascular disease, ultimately contributing to systemic functional decline [15,16,17,18].

A variety of diet-derived bioactives can modulate vascular aging. Polyphenols, such as resveratrol [19], curcumin, quercetin, and fisetin, are capable of alleviating oxidative stress and inflammatory responses [20,21], enhancing endothelial performance, and mitigating the progression of arterial rigidity, according to preclinical and limited clinical studies [19,20,21,22,23,24,25]. Long-chain ω-3 fatty acids can attenuate pro-inflammatory signaling in endothelial cells and atherosclerotic vessels [21], while anthocyanins and other plant-derived compounds, such as corylin, can protect against endothelial senescence and vascular remodeling [26,27]. Enhancing nicotinamide adenine dinucleotide (NAD^+^) levels or activating SIRT1 through nutrition has also been shown to facilitate vascular rejuvenation [28,29]. However, most researchers have focused on disease or injury models. Thus, direct evidence supporting primary vascular aging remains scarce.

Luteolin (Lut), also known as flavone 3′,4′,5,7-tetrahydroxyflavone, is widely distributed in dietary plants such as celery, parsley, and chamomile. Growing evidence suggests its potential role in cardiovascular protection. Previous research has revealed that Lut suppresses inflammatory activation and reduces monocyte adhesion in human microvascular and coronary artery endothelial cells, which are stimulated by interleukin (IL)-1β [30,31]. Under oxidative stress, Lut preserves mitochondrial integrity, limits apoptosis in vein endothelial cells, and restores endothelial function [32]. In co-culture angiogenesis assays, polyphenols, including Lut, reduce angiogenesis while improving endothelial marker profiles [33]. Lut also inhibits vascular smooth muscle cell proliferation and migration [34,35,36]. Most of these studies use acute inflammatory or oxidative stress models rather than aging-specific models and fail to assess canonical senescence endpoints, including senescence-associated β-galactosidase (SA-β-Gal) activity or SASP. As a result, it remains unclear whether or not Lut directly counteracts endothelial senescence.

In addition to its vascular effects, Lut also modulates lipid metabolism and nuclear receptor signaling pathways closely associated with vascular health. In vivo studies have indicated that Lut improves endothelium-dependent relaxation and nitric oxide-mediated function in diet-induced obese mice [37]. It restores the phenotype of perivascular adipose tissue in Goto–Kakizaki rats and improves endothelial function [38]. Lut also attenuates vascular remodeling in pulmonary and systemic hypertension and reduces disease burden in models of vascular calcification, radiation injury, atherosclerosis, and sepsis [35,39,40,41,42,43,44,45]. Recent studies have further revealed that Lut functions as a natural ligand and partial agonist of peroxisome proliferator-activated receptors (PPARs), particularly PPARγ and PPARα. For instance, Lut directly activated PPARγ in ischemia/reperfusion and Alzheimer’s models, thereby suppressing nuclear factor kappa-light-chain-enhancer of activated B cells(NF-κB)-mediated inflammation and restoring mitochondrial homeostasis [46,47]. In metabolic models, Lut enhanced PPARα-dependent fatty acid oxidation and upregulated Carnitine palmitoyltransferase 1 and Pyruvate dehydrogenase kinase 4, improving cardiac energy metabolism and attenuating hypertrophic remodeling [48]. Moreover, other studies demonstrated that polyphenolic compounds including Lut promote PPARα–retinoid X receptor (RXR) signaling, which contributes to lipid clearance and circadian regulation [49]. In parallel, retinoic acid and PPARs form functional heterodimers with RXRs that coordinate lipid oxidation and anti-inflammatory transcriptional programs critical for endothelial homeostasis [50]. Through this retinoic acid–PPAR regulatory network, Lut may influence lipid metabolic remodeling and redox regulation, providing a mechanistic bridge between its vascular-protective and anti-aging effects [51,52]. However, these studies dominantly used disease- or injury-based models, not models of vascular aging as the primary specimens. The studies also rarely incorporate direct senescence measurements or employ integrated omics analyses. One study has reported that *Salvia haenkei* extracts enriched in Lut can extend the lifespan and healthspan of mice and reduce age- and doxorubicin (Dox)-induced senescence. The study also identified that Lut acts as a senomorphic compound, disrupting the interaction between Cyclin-dependent kinase inhibitor 2A and Cyclin-dependent kinase 6 through molecular modeling [53]. This intervention, however, used complex extracts rather than Lut alone and targeted systemic rather than vascular-specific outcomes. Although the current evidence supports the vascular-protective potential of Lut, it provides little or no direct proof for primary vascular aging models, particularly in cross-species settings. Rigorous evaluation focusing on aging-based, vascular-centered models with senescence-specific endpoints is required to more clearly understand the efficacy and mechanism of vascular aging.

Motivated by these gaps, this study examined whether Lut directly attenuates vascular senescence and identified mechanisms. In the study, we used a Dox-induced senescence model in canine and feline vascular endothelial cells, along with senescence-accelerated mouse prone 8 (SAMP8) cohorts (young, aged, and aged treated with Lut for 8 weeks). In all models, we measured canonical senescence endpoints (i.e., SA-β-Gal activity and SASP cytokines), pro-repair and antioxidant indices, and vascular structural and biomechanical parameters. Integrated transcriptomic and metabolomic analyses were employed to define Lut-related pathways, with emphasis on retinoic acid–peroxisome proliferator-activated receptor/retinoid X receptor (PPAR/RXR) signaling and lipid metabolic reprogramming.

## 2. Materials and Methods

### 2.1. Materials and Reagents

Lut (L812409, purity ≥ 98%) was purchased from Macklin Biochemical Co., Ltd. (Shanghai, China) and Dox (D107159, ≥98%) from Aladdin Reagent Co., Ltd. (Shanghai, China). Lut was dissolved in dimethyl sulfoxide (DMSO, purity ≥ 99.90%, Solarbio technology Co., Ltd., Beijing, China) and diluted in minimum essential medium (MEM) (phenol red-free, Procell, Wuhan, China) through dropwise addition. The final DMSO concentration was kept constant at 0.2% in all treatments.

### 2.2. Cell Viability Assay

The Cell Counting Kit-8 (CCK-8) assay was used to evaluate cell viability. Canine vascular endothelial cells (cVECs) and feline vascular endothelial cells (fVECs) were kindly provided by the Global Pet Cell Resource Center (PETCC) (Tianjin, China). Cells were maintained in modified Dulbecco’s Modified Eagle Medium (M3 formulation) enriched with 12% fetal bovine serum (Gibco, Waltham, MA, USA), 100 U/mL penicillin (Gibco, Waltham, MA, USA), and 100 µg/mL streptomycin (Gibco, Waltham, MA, USA) under standard conditions (37.5 °C, 5% CO_2_-humidified atmosphere). Cells were inoculated into 96-well plates at 1 × 10^4^ cells/well. After 24 h, cells were treated with serially diluted Lut or Dox for an additional 24 h. Following aspiration of the medium, cells were exposed to 110 µL of MEM containing 10% CCK-8 reagent (Beyotime, Shanghai, China) and incubated for 2 h. The absorbance was then assessed at 450 nm with a microplate reader (SpectraMax, Molecular Devices, San Jose, CA, USA). Untreated cells cultured in plain MEM were used as a negative control (NC), and cells exposed to 0.2% DMSO in MEM were used as a vehicle control. Dox at 70–85% viability was used to induce senescence.

### 2.3. SA-β-Gal Activity Assay

The anti-senescent effect of Lut was assessed based on SA-β-Gal activity determined using the p-nitrophenyl-β-D-galactopyranoside (pNPG) method. Since SA-β-Gal staining is unsuitable for accurate quantification due to apoptotic or detached cells, enzymatic activity was quantitatively assessed, with staining used for morphological visualization only. cVECs and fVECs were seeded in 96-well plates (1 × 10^4^ cells/well). Cells were treated with a 1:1 mixture of Dox and Lut (the concentration of Lut was 0.625–2.5 µM, whereas that of Dox was 0.8 µM for cVECs and 1.6 µM for fVECs). Cells treated with Dox alone served as the senescence control. Cells were incubated with 100 µL of each treatment per well for 24 h, followed by centrifugation at 1000× *g* for 5 min to discard the supernatant. 20 µL M-PER mammalian cell lysis buffer (Thermo Scientific, Waltham, MA, USA)) was added to each well for lysis. Samples were treated with the pNPG working solution (Solarbio, Beijing, China) and incubated at 37 °C for 3 h. A colorimetric developer was then added, and the incubation was continued for another 12 h. After 12 h, an absorbance at 400 nm, which reflects the SA-β-Gal activity, was measured. The sample in each condition was prepared in a minimum of six replicates.

For visualization, SA-β-Gal staining was performed using a Beyotime kit (Shanghai, China). After treatment, cells were fixed in 100 µL fixative solution for 15 min, followed by three washes with PBS, and then stained with 100 µL X-Gal solution at 37 °C for 18 h. Stained cells were visualized under an inverted microscope.

### 2.4. Evaluation of Cellular Senescence Markers

#### 2.4.1. Assessment of SASP

cVECs and fVECs were seeded into 6-well plates at a density of 2 × 10^5^ cells/well. Senescence was induced with Dox (0.8 µM for cVECs; 1.6 µM for fVECs). Cells co-treated with 0.625 µM Lut were designated as the intervention group, whereas untreated cells were used as the normal control (NC). After 24 h of treatment, the culture medium was centrifuged at 1000× *g* for 5 min to separate the supernatant (Eppendorf 5702 R centrifuge, Hamburg, Germany). M-PER lysis buffer (Thermo Scientific, Waltham, MA, USA) was used to prepare cell lysates.

Levels of IL-1β, tumor necrosis factor (TNF)-α, IL-6, IL-8, matrix metalloproteinase (MMP)-1, MMP-3, and monocyte chemoattractant protein-1 (MCP-1) were quantified using canine- or feline-specific ELISA kits (Mlbio, Shanghai, China). The reaction was terminated using concentrated sulfuric acid, and an absorbance at 450 nm was measured.

#### 2.4.2. Quantification of Angiogenic Repair Factors

The concentration of vascular endothelial growth factor (VEGF) was quantified using ELISA kits (Mlbio, Shanghai, China) from the collected cell media. The level of basic fibroblast growth factor (bFGF) was measured similarly. The procedure has been described in the SASP analysis.

#### 2.4.3. Oxidative Stress Assessment

The whole-cell lysates were measured to assess oxidative stress. Malondialdehyde (MDA) levels were assessed using a thiobarbituric acid-based kit (Beyotime, Shanghai, China) with absorbance measured at 532 nm. Superoxide dismutase (SOD) activity was evaluated with a WST-8-based method, with absorbance measured at 450 nm. NAD^+^ content was detected using an enhanced NAD^+^/NADH kit (Beyotime, Shanghai, China) through sequential incubation with ethanol dehydrogenase, followed by the addition of a colorimetric reagent. Protein concentrations were quantified using the BCA kit (Thermo Scientific, Waltham, MA, USA).

### 2.5. Early Senescence Mouse Model

#### 2.5.1. Experimental Design

The anti-vascular-aging effect of Lut was further evaluated in SAMP8 mice. The SAMP8 strain was provided by the Laboratory Animal Center, Peking University Health Science Center (Beijing, China). Experimental manipulations complied with institutional guidelines and were authorized by the Institutional Animal Care and Use Committee of Hangzhou INCH Biotech (Approval No. INCH-IACUC-2025-03-084, approved on 14 March 2025). All animal experiments were conducted at the Laboratory Animal Center of Zhejiang University (Hangzhou, China), starting in April 2025. Mice were randomly assigned to three groups (*n* = 10 males/group): 1-month-old SAMP8 mice as the young control (YC), 7-month-old SAMP8 mice as the aging control (OC), and 7-month-old SAMP8 mice receiving oral Lut as the intervention group (OC + Lut). After a 7-day acclimatization, mice were maintained at 22 ± 2 °C and a 12 h light/dark cycle, with food and water available ad libitum. Lut was suspended in saline and administered daily by oral gavage at 10 mg/kg for 8 weeks [37,41,42]. Control groups received equivalent volumes of saline. Body weight was recorded weekly. Physiological parameters were assessed 24 h after the final dose. Mice received intraperitoneal anesthesia with 50 mg/kg pentobarbital sodium. The blood was collected from the orbital sinus. Serum was isolated by allowing samples to stand for 2 h and centrifuging at 2000 rpm for 20 min, after which mice were euthanized by cervical dislocation. The abdominal aorta, heart, liver, lungs, and kidneys were rapidly excised, weighed, snap-frozen, and stored at −80 °C until further analysis.

#### 2.5.2. Behavioral Test

Starting from week 4 (day 28) of gavage, motor performance was evaluated weekly using the rotarod fatigue test and balance beam test. Prior to the rotarod test, mice were allowed to acclimate to the testing environment for 30 min and then placed on the rotating rod (initial speed: 4 rpm; acceleration: 0.2 rpm/s, reaching a maximum of 40 rpm). The latency to fall was recorded, with a fall defined as either full-body contact with the base sensor or passive rotation for three consecutive turns. Motor coordination was evaluated using the balance beam test. Mice were placed at one end of the beam, and the time taken to cross the beam was recorded. Each mouse was tested three times, and the values were averaged for analysis.

#### 2.5.3. Physiological Measurements

Vital signs, such as systolic/diastolic blood pressure, heart rate, respiratory rate, and blood oxygen saturation, were recorded 24 h after the final gavage (day 56). Mice were gently restrained in a soft mouse holder for noninvasive monitoring. Blood pressure and heart rate were measured using a tail-cuff system (BP-2010A, Softron, Beijing, China), whereas oxygen saturation and respiratory rate were assessed using a pulse oximeter for small animals (MouseOx Plus, STARR Life Sciences, Oakmont, PA, USA).

#### 2.5.4. Serum Senescence Markers

Serum levels of IL-6, MMP-1, MMP-3, and lipofuscin-binding protein were quantified using mouse-specific ELISA kits (Mlbio, Shanghai, China). Glutathione peroxidase (GPX) activity was determined with a 5,5′-dithiobis (2-nitrobenzoic acid)-based colorimetric assay kit (Solarbio, Beijing, China) by measuring absorbance at 412 nm.

#### 2.5.5. Vascular Tensile Strength

The midsection of the abdominal aorta was isolated (five samples were randomly selected from each group). The vessel was mounted in physiological saline, secured with microclamps at both ends, and then subjected to longitudinal tension at a constant stretching speed of 200 mm/min using a micromechanical testing system (Oberko, Wuhan, China). The peak tensile force was recorded.

#### 2.5.6. Hematoxylin and Eosin (HE) Staining

Paraffin-embedded sections of the abdominal aorta, heart, and liver were subjected to HE staining. Three samples were randomly selected from each group. Tissue samples underwent graded ethanol dehydration, xylene clearing, and paraffin embedding. Transverse sections were prepared using a rotary microtome (RM2235, Leica Microsystems, Shanghai, China). Staining was performed using hematoxylin and eosin (HJ-H0750-4, Hongzijiahua, Beijing, China) according to standard protocols, which included deparaffinization, rehydration, nuclear and cytoplasmic staining, dehydration, and mounting. Microscopic images were captured, and morphological evaluation was conducted using KF-Viewer software (version 1.7.0.21, KFBIO, Ningbo, China). For quantitative assessment, vascular medial thickness was measured at a minimum of 40 evenly spaced points.

### 2.6. RNA Sequencing

Transcriptomic profiling was performed on canine vascular endothelial cells (cVECs) and arterial tissues from SAMP8 mice to investigate underlying molecular mechanisms. For RNA preparation, cells and tissues were homogenized in liquid nitrogen, followed by chloroform separation and isopropanol precipitation. RNA was quality-checked and library construction was performed (details are provided in the Supplementary Material). Final libraries were amplified by PCR and sequenced on the Illumina platform (NovaSeq 6000, Novogene, Beijing, China).

Alignment of raw reads to the corresponding reference genomes was performed using HISAT2 (v2.2.0): *Canis familiaris* (Ensembl 68, CanFam3.1) and *Mus musculus* (Ensembl 92, GRCm38). Reference indices were generated before alignment. FeatureCounts (v2.0) was applied to calculate gene-level read counts and FPKM values, followed by principal component analysis (PCA) on the FPKM profiles. DESeq2 (v1.42.0) was used to identify differentially expressed genes (DEGs). Adjusted *p*-values (padj) were calculated using the Benjamini–Hochberg correction. DEGs were considered with |fold change (FC)| ≥ 1.2 and padj ≤ 0.05.

The gene set enrichment analysis (GSEA) was conducted using the standalone GSEA software (v3.2). Genes were ranked using the Signal2Noise metric to assess expression differences across groups. Enrichment scores (ESs) were computed via permutation testing, with normalized enrichment scores (NESs) adjusting for gene set size. False discovery rates (FDRs) were estimated using the GSEA algorithm.

### 2.7. Untargeted Metabolomics of cVECs

To investigate treatment-related metabolic alterations in cVECs, untargeted metabolomic profiling was performed. After treatment, cVECs were digested with trypsin–EDTA (Gibco, USA) for 2 min and then neutralized with PBS. Cell pellets were obtained by centrifuged at 1000× *g* for 5 min (at 4 °C, Eppendorf 5702 R centrifuge, Hamburg, Germany). Samples were sent to Novogene (Beijing, China) for analysis using a Liquid Chromatography–Tandem Mass Spectrometry (LC–MS/MS) platform (Thermo Fisher Scientific, Waltham, MA, USA). Detailed analytical methods are described in the Supplementary Materials.

### 2.8. Statistical Analysis

All data are presented as mean ± standard deviation (SD). Each group contained at least four replicates, and CCK-8 assays were performed with a minimum of 12 replicates per condition. All statistical evaluations were carried out utilizing the SPSS software (version 26, IBM Corp., Armonk, NY, USA). Group differences were determined using one-way analysis of variance (ANOVA). Prior to multiple comparisons, variance homogeneity was evaluated. Tukey’s post hoc test was employed for datasets with equal variances, whereas the Games–Howell test was applied otherwise. Results with *p* < 0.05 were considered statistically significant.

## 3. Results

### 3.1. Lut’s Effect on Vascular Endothelial Cell Viability

**Scheme 1:**
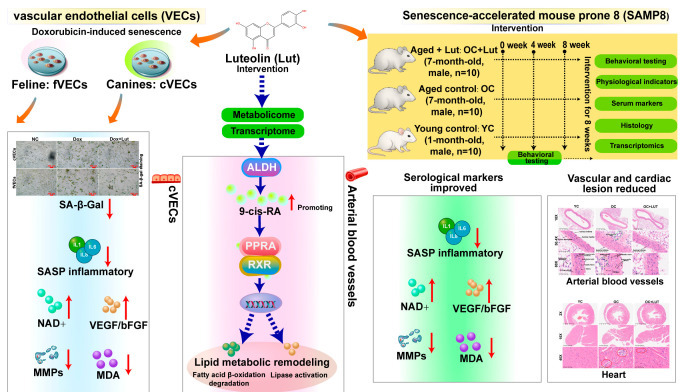
Experimental design and study overview. This schematic diagram illustrates the overall experimental approach, including the intervention with luteolin (Lut) and the different experimental models used in this study.

**Figure 1:**
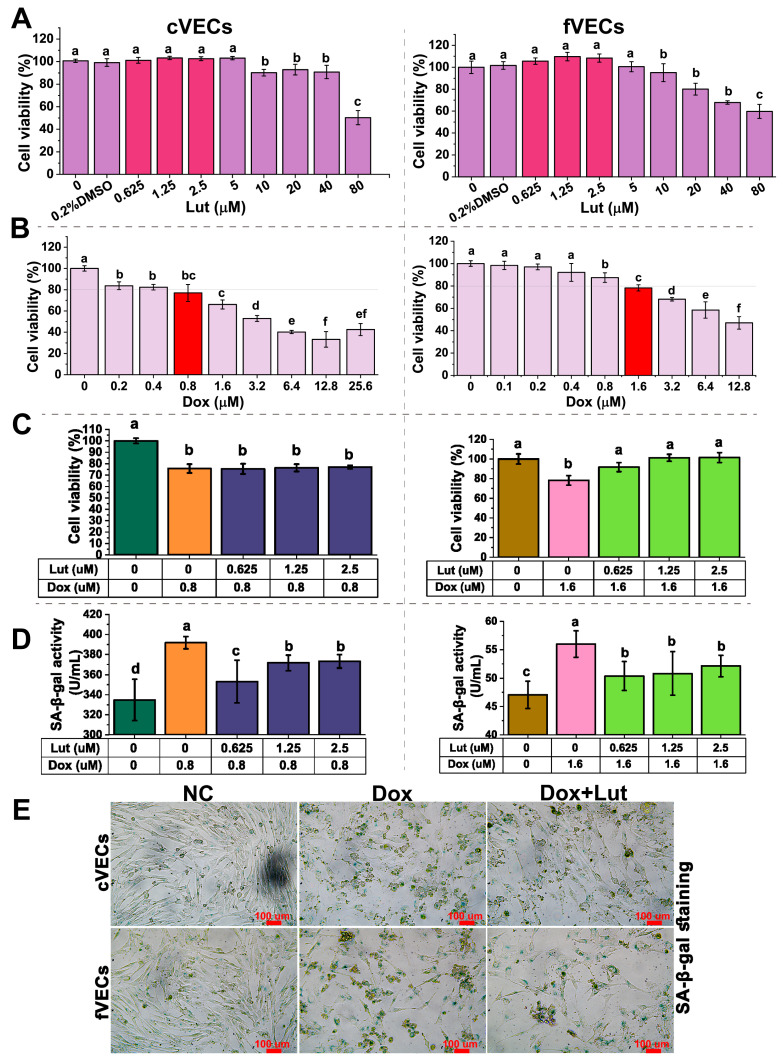
Effects of Lut on cell viability and senescence in vascular endothelial cells. (**A**) Dose–response analysis of Lut (0.04–80 µM) on cell viability in canine vascular endothelial cells (cVECs) and feline vascular endothelial cells (fVECs), assessed by CCK-8 assay (*n* ≥ 12). (**B**) Cell viability after treatment with different concentrations of doxorubicin (Dox; 0.1–25.6 µM) in cVECs and fVECs (*n* ≥ 12). (**C**) Protective effects of Lut (0.625–2.5 µM) against Dox-induced cytotoxicity (0.8 µM in cVECs; 1.6 µM in fVECs) as measured by cell viability (*n* ≥ 12). (**D**) Senescence-associated β-galactosidase (SA-β-gal) activity in cVECs and fVECs under Dox treatment with or without Lut co-treatment (*n* ≥ 10). (**E**) Representative SA-β-gal staining images showing cellular senescence in NC, Dox, and Dox + Lut (0.625 µM) groups in both cVECs and fVECs (*n* ≥ 4). Scale bar = 100 μm. Data are shown as mean ± SD. Distinct lowercase letters above the bars denote significant differences among groups (*p* < 0.05).

As shown in Scheme 1, the overall study design is outlined. We first conducted experiments based on cVECs and fVECs to evaluate the effects of Lut on vascular endothelial cells. Figure 1A shows that Lut at concentrations of 0.625–5 µM exerted no cytotoxic effect on either cVECs or fVECs, while a modest but statistically non-significant increase in cell viability was noted. By contrast, concentrations ≥ 10 µM significantly reduced cell viability. To evaluate the anti-senescence effects of Lut while ensuring both non-toxicity and physiological relevance of the compound, a lower non-toxic range of 0.625–2.5 µM was selected for subsequent experiments.

To establish a senescence model, Dox was applied to induce cytotoxic stress. As shown in Figure 1B, Dox reduced the viability of cVECs as the concentration increased. At 0.8 µM, it decreased viability to approximately 75% of NC (*p* < 0.05). In fVECs, 1.6 µM Dox significantly reduced cell viability to ~80% of NC (*p* < 0.05). Based on these dose–response experiments, it was evident that the two endothelial cell types exhibited different sensitivities to Dox. Concentrations of 0.8 µM for cVECs and 1.6 µM for fVECs consistently and reproducibly reduced cell viability by approximately 20–25%, sufficient to induce early endothelial senescence without causing irreversible cytotoxicity. Therefore, these concentrations were selected for establishing the Dox-induced senescence model.

The cotreatment with Lut and Dox was then evaluated (Figure 1C). In cVECs, Lut (0.625–2.5 µM) had no significant effects on Dox-induced cytotoxicity. However, Lut could alleviate Dox-induced cytotoxicity and promote cell proliferation in fVECs.

### 3.2. Lut Reduced SA-β-Gal Activity

As illustrated in Figure 1D, Dox notably raised SA-β-Gal activity levels in both cVECs and fVECs by approximately 17% and 19%, respectively, compared to NC (*p* < 0.05). Conversely, Lut treatment (0.625–1.25 µM) markedly attenuated this increase. At 0.625 µM, Lut reduced SA-β-Gal activity in both cell types by ~10% (*p* < 0.05). X-Gal staining was further applied to visualize cellular senescence (Figure 1E). In the NC group, only a few SA-β-Gal-stained cells were detected in both cVECs and fVECs. Dox-treated cells showed reduced adherence, shrinkage, and markedly enhanced SA-β-Gal staining. Co-treatment with 0.625 µM Lut alleviated these changes by improving adhesion and attenuating the staining intensity of SA-β-Gal positivity.

### 3.3. Lut Reduced Levels of SASP Markers

**Figure 2:**
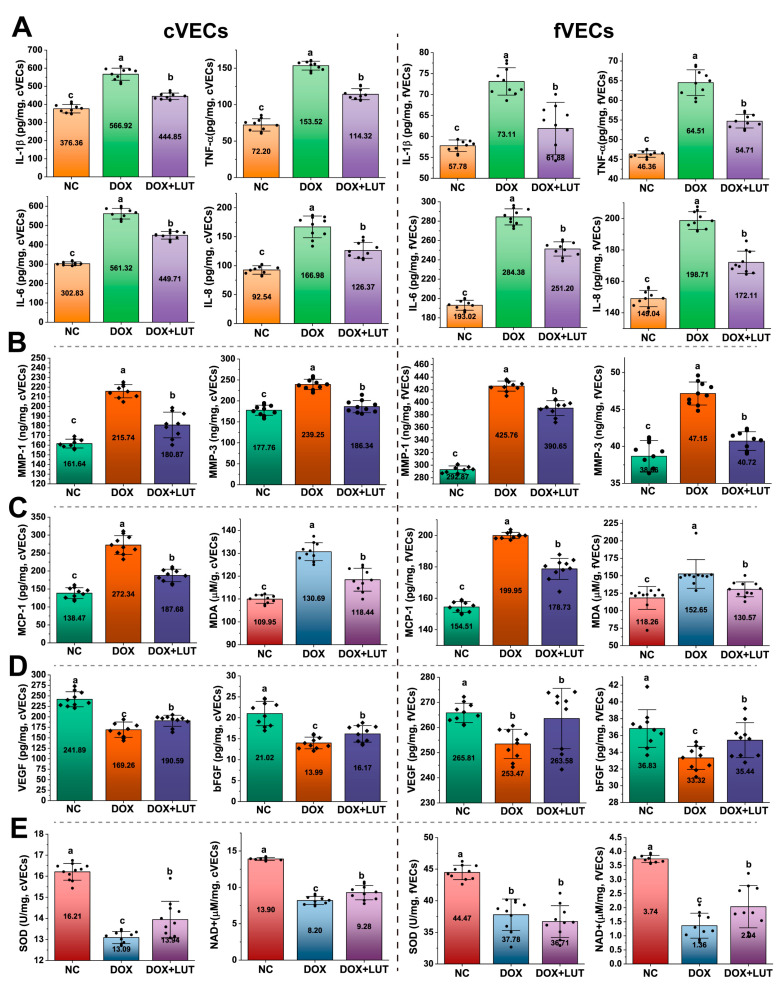
Effects of Lut on oxidative stress and energy metabolism in vascular endothelial cells. cVECs and fVECs were treated with Dox (0.8 µM for cVECs; 1.6 µM for fVECs) with or without 0.625 µM Lut for 24 h. (**A**) Levels of inflammatory SASP markers in culture supernatants, including interleukin (IL)-1β, tumor necrosis factor (TNF)-α, IL-6, and IL-8. (**B**) Levels of matrix metalloproteinase (MMP)-1 and MMP-3. (**C**) Monocyte chemoattractant protein-1 (MCP-1) levels in supernatants and malondialdehyde (MDA) content in cell lysates. (**D**) Secretion of vascular endothelial growth factor (VEGF) and basic fibroblast growth factor (bFGF). (**E**) Intracellular superoxide dismutase (SOD) activity and nicotinamide adenine dinucleotide (NAD^+^) levels. NC: untreated control; Dox: Doxorubicin only; Dox + Lut: Dox combined with 0.625 µM Lut. Data are presented as mean ± SD (*n* ≥ 10). The numerical values in the middle of each bar denote the mean of replicates. Distinct lowercase letters above the bars denote significant differences among groups (*p* < 0.05).

To further assess the effect of Lut on SASP, 0.625 µM Lut was applied in Dox-induced senescent cVECs and fVECs. Dox exposure led to a significant elevation of TNF-α, IL-1β, IL-6, and IL-8 in both cVECs and fVECs, as illustrated in Figure 2A (*p* < 0.05). Co-treatment with Lut led to a significant reduction in the levels of these cytokines (*p* < 0.05). Similarly, Figure 2B,C show that Dox markedly elevated the levels of MMP-1, MMP-3, MCP-1, and MDA in both cVECs and fVECs (*p* < 0.05), while the co-treatment with Lut significantly decreased them (*p* < 0.05).

### 3.4. Lut Enhanced Angiogenic and Antioxidant Responses

As shown in Figure 2D, Dox significantly suppressed the secretion of VEGF and bFGF in both cVECs and fVECs (*p* < 0.05), and the co-treatment with Lut restored the secretion (*p* < 0.05). In Figure 2E, Dox also reduced intracellular SOD activity and NAD^+^ levels (*p* < 0.05) in both cell types. Lut co-treatment significantly elevated both markers in cVECs and increased NAD^+^ levels in fVECs (*p* < 0.05), although the elevation of SOD activity in fVECs was not statistically significant.

### 3.5. Effects of Lut on Physiological and Behavioral Parameters in Aged SAMP8 Mice

**Figure 3:**
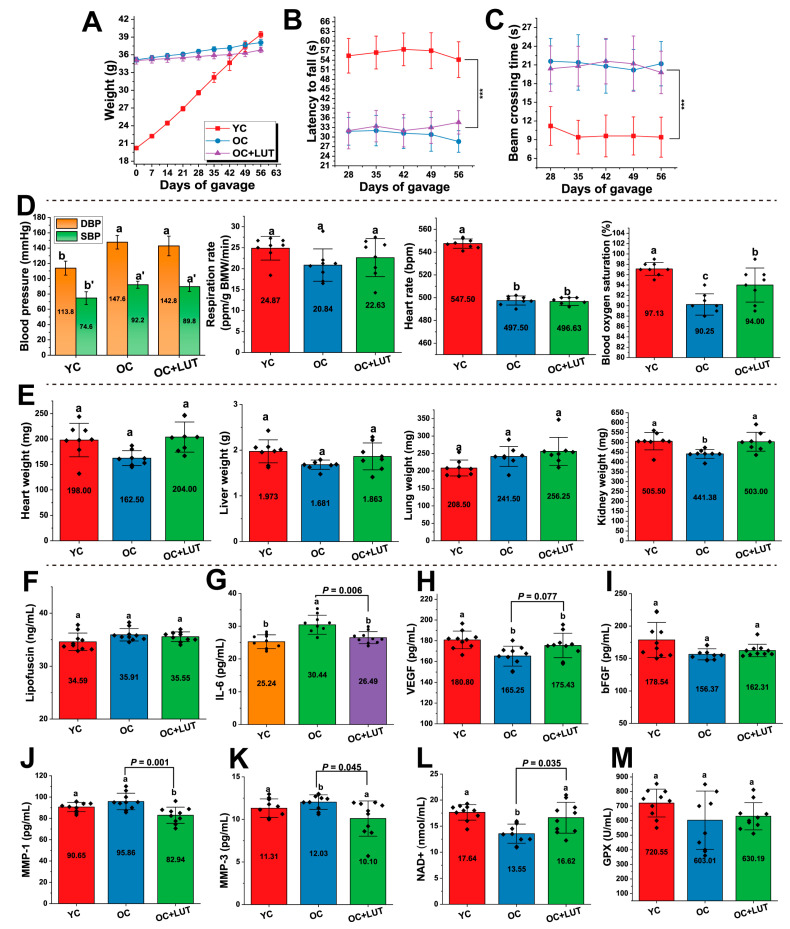
Effects of Lut on physiological, behavioral, and serum parameters in senescence-accelerated SAMP8 mice. Young control (YC): 1-month-old non-senescent SAMP8 mice; old control (OC): 7-month-old aged SAMP8 mice; OC + Lut: 7-month-old SAMP8 mice treated with Lut (10 mg/kg, gavage for 8 weeks). Male mice, *n* = 10 per group. (**A**) Body weight over the gavage period. (**B**) Rotarod performance: latency to fall measured weekly from week 4 of gavage. (**C**) Balance beam test: time to cross the beam measured weekly from week 4 of gavage. (**D**) Terminal physiological parameters, including systolic/diastolic blood pressure (SBP/DBP), respiratory rate (breaths/min per body weight), heart rate (bpm), and blood oxygen saturation (%). (**E**) Terminal organ weights: heart, liver, lung, and kidney. (**F**–**M**) Serum levels of lipofuscin (**F**), IL-6 (**G**), VEGF (**H**), bFGF (**I**), MMP-1 (**J**), MMP-3 (**K**), NAD^+^ (**L**), and GPX (**M**). Data are presented as mean ± SD. The numerical values in the middle of each bar denote the mean of replicates. Distinct lowercase letters above the bars denote significant differences among groups (*p* < 0.05). In panels B and C, *** indicate statistically significant differences compared with the YC and OC groups at *p* < 0.001.

As shown in Scheme 1, an 8-week Lut gavage intervention was conducted in SAMP8 mice to evaluate its in vivo anti-aging effects. After 8 weeks of gavage, the body weights of OC and YC mice were not significantly different (*p* > 0.05) (Figure 3A). Rotarod performance was evaluated weekly beginning in week 4 (Figure 3B). Latency to fall of OC mice was significantly lower compared to YC mice (*p* < 0.05), which is indicative of impaired motor coordination. Treatment with Lut increased the latency, but the increase was not significant (*p* > 0.05). The balance beam test (Figure 3C) indicated that crossing times of OC mice were significantly prolonged compared to YC mice (*p* < 0.05). By contrast, the crossing times of OC + Lut mice were reduced compared to OC mice; however, the reduction was not statistically significant (*p* > 0.05). Terminal physiological measurements are shown in Figure 3D. Compared with YC, OC mice exhibited significantly higher blood pressure and lower heart rate and blood oxygen saturation (all *p* < 0.05). Blood oxygen saturation of Lut-treated mice (OC + Lut) was significantly higher compared to OC mice (*p* < 0.05), but their blood pressure and heart rate were not significantly different (*p* > 0.05). Organ weights are summarized in Figure 3E. There were no significant differences in the weights of the heart, liver, and lungs across the three groups (*p* > 0.05). In contrast, the kidney weight in OC mice was notably lower than that in the YC and OC + Lut groups (*p* < 0.05). This suggests that Lut treatment could partially restore the kidney weight.

### 3.6. Lut Improved Serum Aging-Related Markers in Aged SAMP8 Mice

As shown in Figure 3G,J,K, serum levels of SASP markers (IL-6, MMP-1, and MMP-3) were significantly higher in OC mice (*p* < 0.05) but notably decreased in OC + Lut-treated mice compared to YC mice (*p* < 0.05). As illustrated in Figure 3H,I,L–M, levels of VEGF, bFGF, NAD^+^, and GPX were lower in OC mice than in YC mice. The reduction in VEGF (Figure 3H) and NAD^+^ (Figure 3L) was statistically significant (*p* < 0.05), as was the increase observed following Lut treatment (*p* < 0.05). An increase in bFGF (Figure 3I) and GPX (Figure 3M) levels was observed in the OC + Lut group; however, the increase was not statistically significant (*p* > 0.05). These trends were consistent with those observed in vitro in both cVECs and fVECs, indicating a potential protective effect of Lut against vascular aging across species.

### 3.7. Lut Improved Vascular Structure and Mechanical Properties in Aged SAMP8 Mice

**Figure 4:**
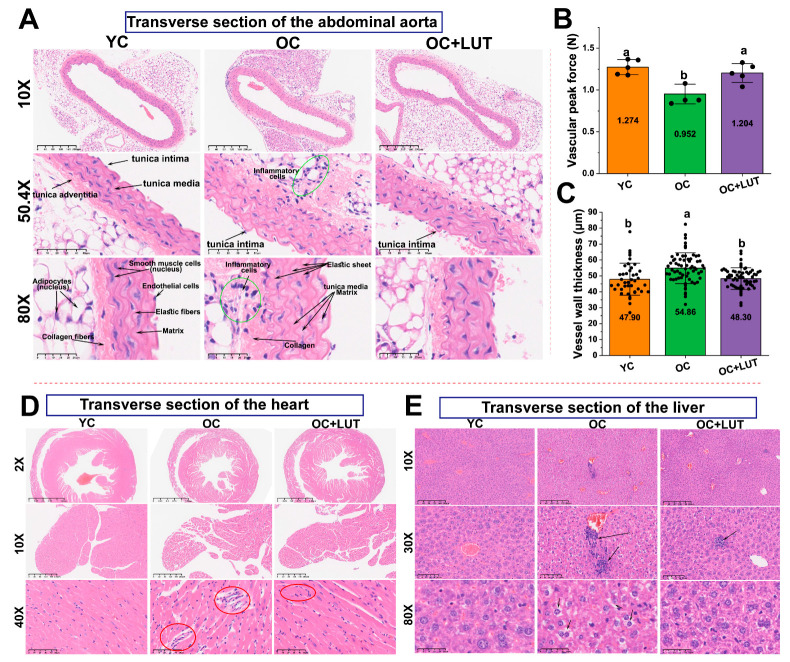
Histological evaluation of the abdominal aortas, heart and liver in aged SAMP8 mice after Lut intervention. (**A**) Representative hematoxylin and eosin (HE)-stained sections of abdominal aortas under 10×, 50.4×, and 80× magnification. (**B**) Peak vascular tensile force of abdominal aortas (*n* = 5 per group). (**C**) Tunica media thickness of abdominal aorta walls (*n* = 3 per group, more than 10 points were sampled for each tissue). (**D**) Representative HE staining of heart sections at 2×, 10×, and 40× magnifications. Red circles indicate areas of inflammatory cell infiltration. (**E**) Representative HE staining of liver sections at 10×, 30×, and 80× magnifications. Arrows indicate areas of inflammatory cell infiltration. YC, young control; OC, aged control; OC + LUT, aged SAMP8 mice treated with Lut for 8 weeks. Data are shown as mean ± SD. The numerical values in the middle of each bar denote the mean of replicates. Distinct lowercase letters above the bars denote significant differences among groups (*p* < 0.05).

H&E staining of abdominal aorta sections (Figure 4A) revealed that OC mice exhibited disorganized vascular layers, with marked inflammatory cell infiltration around the adventitia and adipose tissue (50.4× and 80×), increased collagen deposition (50.4× and 80×), and significantly thickened tunica media. At 80× magnification, the thickening of elastic lamellae and matrix accumulation were prominent. In contrast, OC + Lut mice had improved structural integrity, reduced inflammation, thinner medial layers, and lower collagen and matrix accumulation, similar to the YC group.

Quantitative analysis demonstrated that the vascular peak force of OC mice was significantly lower than that of YC mice, while tunica media thickness was markedly greater (Figure 4B,C, both *p* < 0.05). Lut treatment significantly reversed these changes by increasing vascular tensile strength and reducing medial thickness (*p* < 0.05).

### 3.8. Lut Ameliorated Age-Related Cardiac and Hepatic Histological Alterations in SAMP8 Mice

H&E staining of heart sections (Figure 4D) at both low (2×) and higher (10×) magnifications revealed that, compared to YC mice, OC mice exhibited disorganized myocardial fibers and increased interstitial vacuolation. These features are indicative of myocardial degeneration. In contrast, OC + LUT mice had improved cardiac architecture and reduced vacuolation. Observed at higher magnification (40×), OC mice exhibited widened inter-bundle spaces and prominent focal inflammatory infiltration (red circles). These pathological changes were markedly alleviated in the OC + LUT group, which showed reduced inflammatory cells and preserved myocardial structure.

Similarly, the observations of liver sections (Figure 4E) at 30× magnification demonstrated that OC mice had disorganized hepatic cords, sinusoidal dilation, substantial perivenular inflammatory infiltration (black arrows), and focal hemorrhage, along with nuclear pyknosis in some hepatocytes. At higher magnification (80×), hepatocytes of OC mice showed vacuolated cytoplasm with reduced granularity, indicative of potential glycogen depletion and aging-associated hepatic damage. In contrast, OC + LUT mice showed reduced inflammatory infiltration and improved hepatic architecture and absence of evident hemorrhagic foci. These results suggest that LUT can protect against age-related structural damage in the heart and liver of SAMP8 mice.

### 3.9. Activation of the p53 Signaling Pathway in Dox-Induced cVECs

To elucidate the mechanism by which Lut reduces the expression of Dox-induced senescence-related genes in cVECs, mRNA sequencing was performed. The PCA in Figure S1A revealed distinct transcriptomic profiles among the three groups, showing that Lut significantly altered the gene expression pattern. The Venn diagram (Figure S1B) revealed 3064 DEGs between Dox and NC, 829 DEGs between Dox + Lut and Dox, with 634 overlapping genes. Enrichment analysis of DEGs between Dox and NC using the Kyoto Encyclopedia of Genes and Genomes (KEGG) pathways (Figure S1C) revealed that the aging-related pathways, including p53 signaling and Hippo signaling pathways, were significantly activated, while cell cycle and autophagy pathways were suppressed. This suggests that Dox-induced senescence involves stress and programmed damage responses. GSEA analysis further confirmed the activation of the p53 pathway (Figure S1D).

Heatmap clustering of enriched genes (Figure S1D and Table S1) revealed the activation of key genes in the p53 pathway. Genes involved in cell cycle arrest, including *CDKN1A*, *GADD45A*, *CCNG1*, and *PPM1D*, were markedly upregulated. In response to genotoxic stress, these genes act as canonical p53 downstream effectors to induce G1/S or G2/M checkpoint arrest. The upregulation of these genes indicates that the cell cycle inhibition was triggered in Dox-treated cVECs. In parallel, apoptosis-related genes, including *BAX* (Bax), *CASP3/9* (Caspase-3/9), *PIDD* (PIDD1), *PERP* (Perp), and *APAF1* (Apaf-1), were markedly upregulated, along with oxidative stress regulators *SESN1/2/3* (Sestrins). This implies that Dox triggers p53-mediated apoptosis and senescence in cVECs. These findings collectively support the involvement of Dox-induced cVEC senescence in the activation of cell cycle arrest and apoptosis cascade downstream of the p53 signaling pathway. KEGG annotation (Figure S1E) further illustrated that genes encoding the p53 downstream effectors for DNA repair, cell cycle arrest, apoptosis, and senescence were upregulated.

### 3.10. Lut Activated cAMP Signaling and Lipolytic Pathways in Dox-Induced cVECs

**Figure 5:**
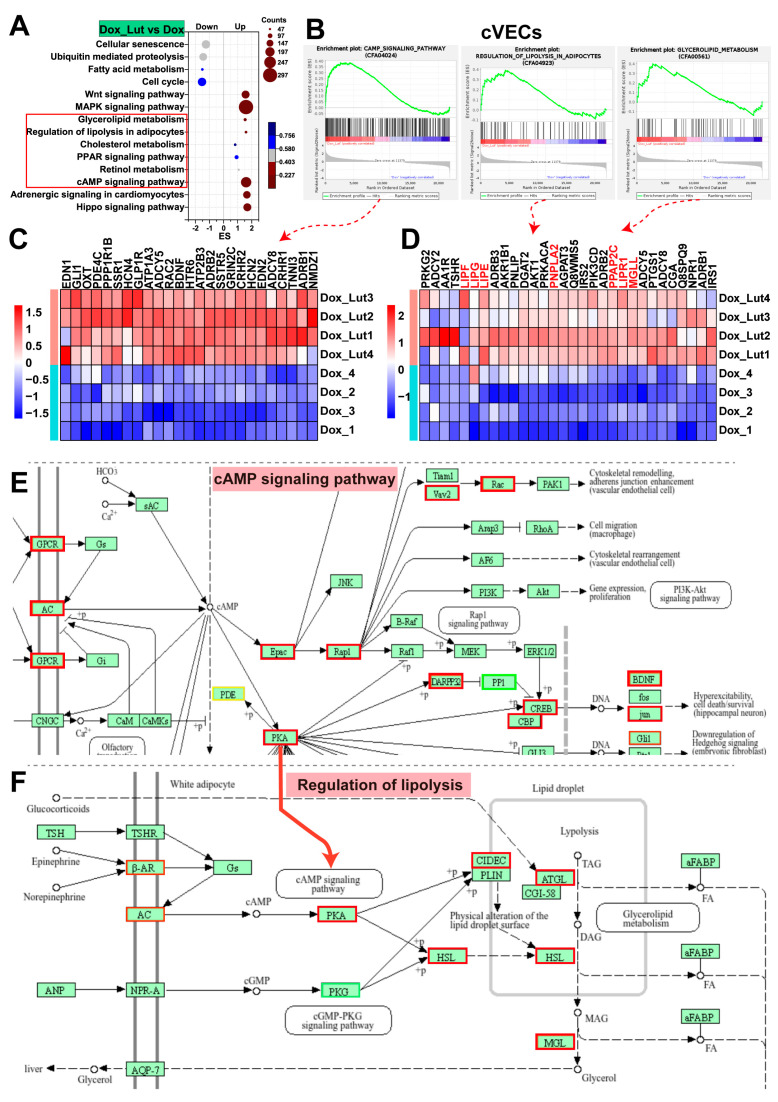
Transcriptomic analysis revealed Lut-induced activation of metabolic and signaling pathways in Dox-treated cVECs. (**A**) KEGG enrichment bubble plot of core pathways regulated by Lut in Dox-induced cVECs (Dox_Lut vs. Dox) based on GSEA. (**B**) GSEA enrichment plots of representative upregulated pathways: cAMP signaling, regulation of lipolysis in adipocytes, and glycerolipid metabolism. (**C**) Hierarchical clustering heatmap of core DEGs in the cAMP pathway. (**D**) Heatmap of shared core DEGs involved in both regulation of lipolysis and glycerolipid metabolism. (**E**,**F**) KEGG pathway maps of (**E**) cAMP signaling and (**F**) regulation of lipolysis, with significantly upregulated genes marked in red boxes and downregulated in green.

Transcription profiling revealed that the treatment with Lut markedly activated the cyclic adenosine monophosphate (cAMP) signaling pathway in Dox-induced cVECs. This was accompanied by the upregulation of lipid catabolic programs, including retinol metabolism, PPAR signaling pathway, cholesterol metabolism, lipolysis, and glycerolipid metabolism (Figure 5A,B). Hierarchical clustering of differentially expressed genes (Table S2) revealed a sequential activation of the signaling pathway involving G-protein coupled receptor (GPCR), adenylyl cyclase (AC), cAMP, and protein kinase A (PKA). Specifically, Lut upregulated multiple GPCRs (*TSHR*, *CRHR1*, *GLP1R*, *DRD1*, *HTR6*, *SSTR5*, and *AA2AR*) and increased the expression of adenylyl cyclases (*ADCY2*, *ADCY5*, and *ADCY8*), indicative of enhanced cAMP production (Figure 5C,E). The downstream PKA branch was also activated, as evidenced by elevated levels of *PRKACA*, *PPP1R1B*, *CREB3*, and *JUN*. This suggests that the cAMP-mediated transcriptional reprogramming was initiated.

Importantly, activation of the cAMP–PKA axis was closely correlated with upregulation of canonical lipolytic enzymes. The levels of *PNPLA2* (ATGL), *LIPE* (HSL), and *MGLL* (MGL) were significantly upregulated (Figure 5D). This represents the core enzymatic cascade responsible for breaking down triacylglycerol (TAG) into diacylglycerol (DAG), monoacylglycerol (MAG), and free fatty acids. These lipases are direct or indirect effectors downstream of PKA activation, consistent with Lut-induced mobilization of stored lipid. Additionally, *LIPG, LIPF,* and *PNLIP,* which encode secreted and tissue-associated lipases, were also upregulated, which is indicative of reinforcement of systemic lipid turnover (Figure 5F). Collectively, these findings indicate that Lut may reprogram lipid metabolism under Dox-induced stress by activating the GPCR–AC–cAMP–PKA cascade and inducing the transcription of the lipolytic enzyme network.

### 3.11. Lut Activated Retinol Metabolism and PPAR Signaling in Dox-Induced cVECs

**Figure 6:**
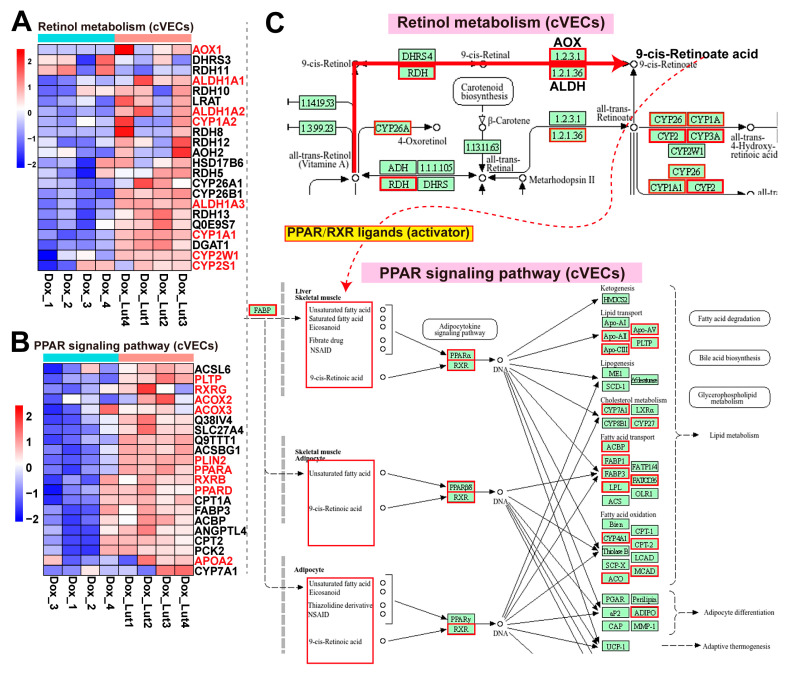
Lut (0.625 μM) activated retinol metabolism and PPAR signaling pathways in Dox-induced (0.8 μM) senescent cVECs. (**A**) Heatmap of representative DEGs in the retinol metabolism pathway comparing Lut + Dox and Dox groups. Expression values were normalized and Z-score-transformed. (**B**) Heatmap of core DEGs within the PPAR signaling pathway, showing transcriptional activation following Lut treatment. (**C**) KEGG pathway maps of retinol metabolism and PPAR signaling. Genes upregulated are marked in red and those downregulated in green, reflecting Lut-induced activation of lipid regulatory pathways in stressed endothelial cells.

Transcriptomic analysis revealed that Lut significantly upregulated the retinol metabolism and PPAR signaling pathways in Dox-induced cVECs (Figure 5A). As shown in the heatmap of differentially expressed genes (Figure 6A), multiple key enzymes involved in retinoid processing were markedly upregulated following Lut treatment. These genes included retinol dehydrogenases (*RDH10*, *RDH11*), retinaldehyde dehydrogenases (*ALDH1A1*, *ALDH1A2*, *ALDH1A3*), and aldehyde oxidase (*AOX1*). This finding suggests the enhancement in sequential oxidation from retinol to retinal and ultimately to retinoic acids, particularly 9-cis-retinoic acid (RA) (Figure 6C). Additionally, significant upregulation of cytochrome P450 family members (*CYP26A1*, *CYP26B1*, *CYP1A1*, *CYP1A2*, *CYP2S1*, and *CYP2W1*) was observed, which is indicative of active catabolism and feedback regulation of retinoic acids. This confirms the presence of a functional retinoid flux under Lut stimulation.

Concomitantly, the PPAR signaling pathway was activated (Figure 6B). Lut induced the expression of canonical PPAR transcription factors, including *PPARA*, *PPARD*, *RXRB*, and *RXRG*, and genes involved in the downstream lipid metabolism such as *ACOX2*, *ACOX3*, *CPT1A*, *CPT2*, *PLTP*, *FABP3*, and *PLIN2* (Figure 6C). Together, these genes mediate fatty acid transport, β-oxidation, and lipid droplet remodeling, reflective of a coordinated transcriptional program for supporting lipid catabolism and metabolic adaptation.

These changes are consistent with the lipid degradation initiated downstream of the Lut-induced cAMP–PKA axis and imply that there is a mechanistic link between retinoic acid accumulation and PPAR activation. Altogether, these findings indicate that, under Dox-induced stress, Lut promotes a transcriptional cascade involved in retinoid metabolism and PPAR signaling, which in turn enhances lipid degradation and oxidative catabolism.

### 3.12. Validation of Lut-Induced Retinoid Activation and Lipid Metabolism Remodeling in cVECs via Metabolomics

**Figure 7:**
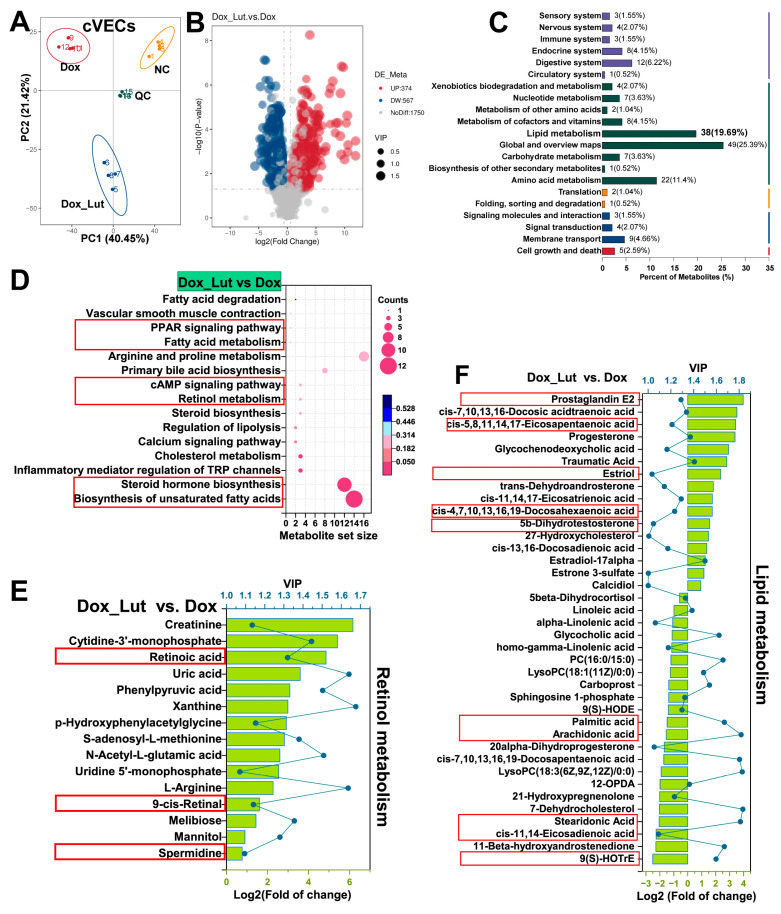
Metabolomic analysis of cVECs treated with 0.625 μM Lut under 0.8 μM Dox-induced senescence. (**A**) PCA displaying clear separation of metabolic profiles between NC, Dox, and Dox + Lut groups (*n* = 4). QC samples were generated by combining equal quantities from all experimental samples, with tight clustering demonstrating good reproducibility and stability. (**B**) Volcano plot of differential metabolites in Dox + Lut vs. Dox. Red and blue dots represent significantly upregulated and downregulated metabolites, respectively (*p* < 0.05). (**C**) KEGG functional classification of differential metabolites, highlighting the dominant enrichment in lipid metabolism (19.69%). (**D**) KEGG pathway enrichment bubble plot of altered metabolites between Dox + Lut and Dox groups. Bubble size reflects pathway metabolite count; color indicates *p* value. (**E**) Log2 fold change in representative metabolites involved in retinol metabolism, including significantly increased retinoic acid and 9-cis-retinal, supporting retinoid activation. (**F**) Differential expression of lipid metabolism-related metabolites, including PUFAs (e.g., EPA and docosadienoic acid), prostaglandins, and steroid hormones (e.g., estriol and 5α-DHT), reflecting enhanced lipid remodeling and degradation.

To validate the effects of Lut intervention on the metabolism of Dox-induced cVECs, untargeted metabolomic analysis was performed. PCA revealed a distinct separation between the Dox and Dox + Lut groups, indicative of clear metabolic divergence (Figure 7A). Volcano plots showed that 374 metabolites were upregulated and 547 were downregulated following Lut treatment (Figure 7B). KEGG classification indicated that 19.69% of the metabolites that were altered were involved in lipid metabolism, and these metabolites dominated the metabolic changes (Figure 7C).

Pathway enrichment analysis (Figure 7D) further confirmed the upregulation of retinol metabolism, PPAR signaling, fatty acid degradation, regulation of lipolysis, unsaturated fatty acid biosynthesis, and cAMP signaling, consistent with the transcriptomic results. Specifically, the levels of several key retinoids, including retinoic acid (FC = 28.986) and 9-cis-retinal (FC = 3.057), were significantly elevated (Figure 7E). This indicates that the conversion from retinol to active retinoids, particularly 9-cis-RA, was enhanced. 9-cis-RA is known as a PPAR/RXR ligand that drives transcriptional activation.

In addition, multiple lipid-related metabolites were differentially regulated (Figure 7F). Among the upregulated metabolites, prostaglandin E2 (PGE2), cis-5,8,11,14,17-eicosapentaenoic acid (EPA), traumatic acid, and glycochenodeoxycholic acid are known to promote lipid catabolism, inflammation resolution, or PPAR activation. Conversely, several lipotoxic or pro-inflammatory lipids, including arachidonic acid (AA), palmitic acid (PA), sphingosine-1-phosphate (S1P), and 9(S)-HODE, were significantly downregulated. This indicates relief of lipotoxicity and oxidative stress. Collectively, these results confirm that Lut activates the retinoid–PPAR axis and induces metabolic reprogramming that enhances lipid degradation, promotes retinoic acid turnover, and supports adaptive stress responses in cVECs.

### 3.13. Lut Activated Retinol/PPAR Signaling and Lipid Metabolism in Vasculature of Aged SAMP8 Mice

To evaluate the in vivo impact of Lut on the vasculature, mRNA from abdominal aorta tissues of aged SAMP8 mice after 8 weeks of oral intervention was sequenced. PCA revealed clear separation among the YC, OC, and OC + Lut groups, which is indicative of distinct transcriptomic profiles (Figure S2A). Compared to YC, the protein processing in the endoplasmic reticulum and N-glycan biosynthesis of aged OC mice were significantly suppressed. In contrast, indicators such as primary immunodeficiency and chemical carcinogenesis-reactive oxygen species were markedly upregulated (Figure S2B). Moreover, age-related lipid-associated pathways involved in non-alcoholic fatty liver disease, and fatty acid elongation were activated, indicative of impaired lipid turnover and metabolic adaptation in aged vasculature.

In contrast, Lut administration significantly reversed the age-associated transcriptomic alterations in vasculature. GSEA revealed that retinol metabolism, PPAR signaling, and cholesterol metabolism were activated, and bile secretion, fat digestion and absorption, and fatty acid degradation were enhanced (Figure S2C). These changes suggest that lipid catabolism and metabolic capacity in senescent vessels were restored. Notably, retinol metabolism, PPAR signaling, and cytochrome P450-mediated drug metabolism were significantly upregulated, consistent with increased clearance of oxidative intermediates and bioactive lipids. Concurrently, immune-senescence-related pro-inflammatory pathways, including T cell receptor signaling, programmed death-ligand 1 (PD-L1) checkpoint, type I diabetes, and NF-κB signaling, were significantly suppressed, an indication of reduced vascular inflammation. Enrichment plots further confirmed Lut activated retinol metabolism, PPAR signaling, and cholesterol metabolism (Figure S2D–F), reinforcing the association between lipid clearance and transcriptional repair.

### 3.14. Lut Caused Vascular Activation of the Retinol/PPAR Axis Across Models and Species

**Figure 8:**
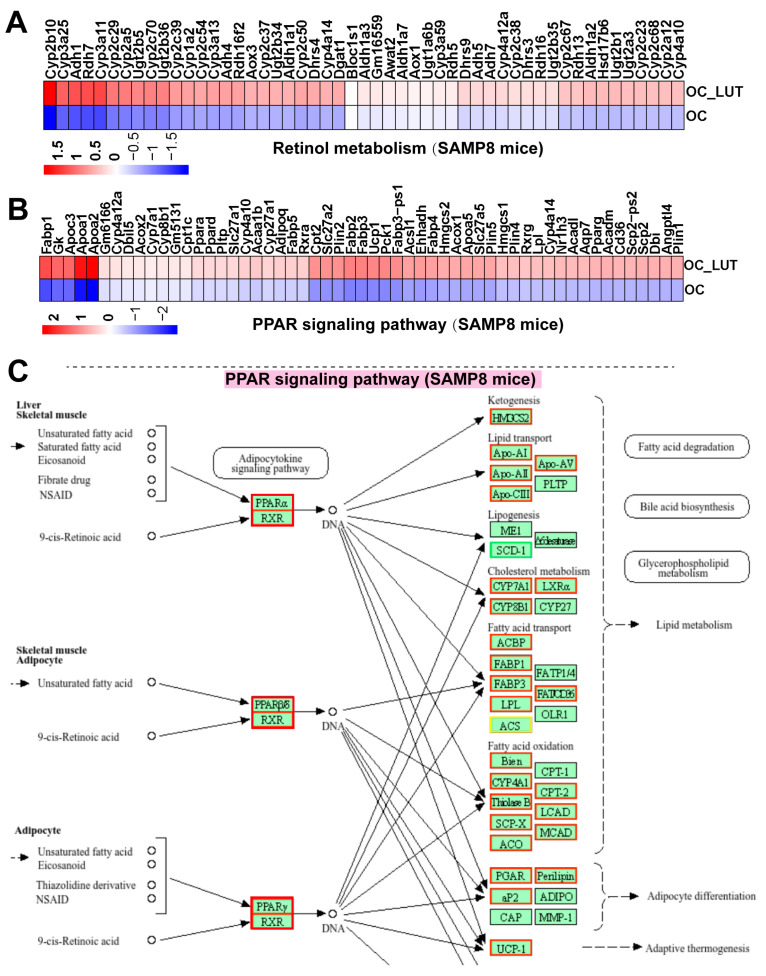
Transcriptomic activation of retinol and PPAR signaling pathways in aged SAMP8 aorta after 8-week oral administration of Lut. (**A**) Heatmap of DEGs in the retinol metabolism between OC_Lut and OC groups. (**B**) Heatmap of DEGs in the PPAR signaling pathway between OC_Lut and OC groups. (**C**) KEGG pathway annotation of PPAR signaling showing upregulated (red border) and downregulated (green border) genes. Key targets include PPARα, PPARδ, PPARγ and RXR heterodimers, with downstream targets involved in lipid metabolism (fatty acid oxidation, transport, and biosynthesis), bile acid metabolism, glycerophospholipid metabolism, adipocyte differentiation, and adaptive thermogenesis.

Consistent with the transcription mechanisms observed in vitro, Lut activated the retinol/PPAR axis in the vascular tissue of aged SAMP8 mice in vivo. Analysis of core genes revealed that key enzymes in the retinol metabolism pathway, including *Rdh10*, *Aldh1a1*, *Aldh1a3*, and *Cyp26a1*, were significantly upregulated, indicating that the synthesis and turnover of 9-cis-RA was enhanced (Figure 8A). Similarly, main regulators of the PPAR signaling pathway, including *Ppara*, *Ppard*, *Pparg*, and *Rxr*, and downstream lipid catabolic genes such as *Lpl*, *Acox1*, *Fabp1/3*, *Cpt1*, and *Ucp1* were upregulated (Figure 8B). The KEGG pathway analysis further revealed activation of genes involved in fatty acid degradation, cholesterol metabolism, bile acid synthesis, and adaptive thermogenesis (Figure 8C). Importantly, these in vivo transcriptional changes in aged SAMP8 mice are consistent with those previously observed in Dox-induced cVECs. This validates that Lut induces the activation of the retinol–PPAR network in canine and murine, as well as in chemically induced and age-associated models, both in vitro and in vivo.

## 4. Discussion

This study revealed that Lut reprogrammed lipid metabolism in aging vasculature by coordinately activating the retinol metabolism–PPAR signaling cascade. Transcriptomic and metabolomic analyses identified a conserved regulatory axis shared between Dox-induced cVECs and aortas of senescence-accelerated SAMP8 mice. Lut significantly enhanced the biosynthesis and accumulation of 9-cis-RA, a key ligand for the PPAR/RXR heterodimer, thereby triggering downstream lipolytic and oxidative gene expression (Scheme 1). The activation was accompanied by marked suppression of inflammatory and immunosenescence-associated pathways. The convergence of transcriptomic and metabolic remodeling was observed across models and species, and this suggests that Lut is a modulator targeting the retinol–PPAR axis during vascular aging.

In the in vitro component of this study, we employed primary mammalian endothelial cells (cVECs and fVECs) because they develop reproducible stress-induced endothelial dysfunction under Dox exposure and display features that are closely aligned with endothelial senescence. These cells preserve the inflammatory and oxidative responsiveness characteristic of adult mammalian endothelium, making them suitable for examining aging-related regulatory pathways. Although human umbilical vein endothelial cells (HUVECs) are highly informative for angiogenic and inflammatory research [30,54], cVECs and fVECs offer a complementary endothelial context that more closely reflects adult vascular physiology. This makes them particularly relevant for investigating pathways associated with age-related endothelial decline. At the organismal level, SAMP8 mice were selected because they are an established model of age-associated vascular deterioration [55,56]. They spontaneously develop progressive arterial stiffness, metabolic dysregulation, and heightened inflammatory activation, allowing vascular aging processes to be captured within a feasible experimental window. Therefore, integrating these complementary systems allows us to assess the effects of Lut across cellular and organismal contexts, rather than relying on model-specific phenomena.

Mechanistically, Lut-induced lipid turnover was linked to activation of the cAMP-PKA signaling axis. Transcriptional enrichment and upregulation of key lipolytic enzymes such as *PNPLA2* and *MGLL* supported this mechanism. This finding aligns with previous reports demonstrating that Lut modulates cAMP signaling in various systems. In a cerebral ischemia/reperfusion model, Lut reduced neuronal injury via transcriptomic reprogramming of cAMP-related pathways [57]. In PC12 cells, Lut activated the cAMP/PKA-cAMP response element-binding protein axis, the effects of which are generally blocked by adenylate cyclase and PKA inhibitors [58]. In microbial systems, flavonoids (including Lut) enhanced cAMP biosynthesis by inhibiting xanthine oxidase activity and reducing oxidative stress [59]. Interestingly, the opposite effect was observed in human embryonic kidney 293 (HEK293) cells, where Lut suppressed odorant-induced cAMP production [60]. This suggests that the impact of Lut on cAMP signaling is dependent on context and cell types. These findings collectively support our results, in which Lut was found to activate the cAMP-PKA signaling pathway in endothelial cells and cooperate with the retinoid–PPAR axis to restore lipid homeostasis during vascular aging.

Transcriptomic and metabolomic analyses consistently demonstrated that Lut treatment activated the retinol metabolic pathway by upregulating key retinol dehydrogenases and aldehyde dehydrogenases, including *Rdh10*, *Aldh1a1*, *Aldh1a3*, and *Cyp26a1*. These enzymes catalyze the critical steps in the synthesis and turnover of 9-cis-RA, whose accumulation enhances retinoid flux, as confirmed by LC-MS analysis. As a transcriptionally active signaling molecule, 9-cis-RA serves as a high-affinity endogenous ligand for RXR, promoting the formation of RXR-PPAR heterodimers and nuclear transactivation of lipid catabolic and mitochondrial functional genes [61,62,63]. This mechanism aligns with previous studies, in which 9-cis-RA was found to induce cholesterol efflux and anti-inflammatory responses in vascular tissues [64,65]. Considering the age-associated decline in RXR signaling and retinoid bioavailability [66,67], the restoration of 9-cis-RA biosynthesis by Lut is considered a crucial regulatory axis for metabolic reprogramming in senescent vasculature. Together, these results indicate that retinoid metabolism acts not merely as a micronutrient supply but as a central transcriptional regulator in vascular rejuvenation. Inducing the activation of this pathway using Lut is a potent strategy for restoring retinoid–PPAR signaling and improving lipid homeostasis in aging vessels.

Transcriptomic analysis revealed that Lut markedly upregulated the expression of *Ppara*, *Pparg*, and *Rxra* and induced canonical PPAR downstream targets, including *Lpl*, *Acox1*, *Cpt1*, and *Ucp1*. These transcriptional changes are indicative of enhanced fatty acid oxidation, thermogenesis, and peroxisomal β-oxidation [68,69,70]. Metabolomic profiling further confirmed the accumulation of pro-lipolytic lipid mediators, such as EPA, and PGE2, and the reduction in AA and S1P. This lipidomic remodeling not only reflects that lipolysis is active, but also suggests the presence of a systemic shift toward anti-inflammatory and pro-resolving lipid signaling [71,72]. The observed activation of PPAR signaling, particularly through the RXR/PPARα axis, implies that Lut orchestrated lipid metabolic reprogramming in aged vasculature by coupling transcriptional control with metabolite remodeling [46]. Previous studies have reported similar observations, where Lut stimulates PPARγ activity and ameliorates vascular dysfunction and insulin resistance in high-fructose-fed rats. These effects are generally blocked by the PPARγ antagonist [47,52]. Moreover, Lut has been shown to suppress vascular inflammation and endothelial adhesion molecule expression by inhibiting NF-κB, which reinforces its role in restoring vascular homeostasis [73]. These findings are consistent with our data, supporting our conclusion that Lut restores lipid clearance and redox balance via PPAR activation. Given the decline in PPAR activity during aging, along with its critical role in lipid metabolism and inflammation resolution [47], mediating PPAR activation using Lut represents a promising strategy for rejuvenating senescent vasculature.

In addition to the cross-species evidence generated from canine, feline, and murine models, multiple studies using human endothelial cells further support the relevance of Lut’s anti-vascular-aging mechanisms. In HUVECs and human microvascular endothelial cells, Lut has repeatedly been shown to attenuate key drivers of endothelial aging—including inflammation, oxidative stress, and mitochondrial injury—through pathways that closely parallel those identified in our study. For example, Lut suppresses TNF-α–induced endothelial activation by inhibiting the protein kinase B/mitogen-activated protein kinase/NF-κB axis, leading to reduced expression of adhesion molecules and inflammatory mediators [30,54], a hallmark event in endothelial senescence. Lut also prevents nitric oxide (NO)-mediated hyperpermeability and exhibits strong NO-radical scavenging activity [54], consistent with the barrier-protective and angiogenic-restoring effects we observed in aging cVECs/fVECs and SAMP8 vasculature. Oxidative stress is a central driver of vascular aging, and multiple human-cell studies demonstrate that Lut protects endothelial cells from H_2_O_2_-induced mitochondrial dysfunction, and apoptosis by suppressing ROS/NF-κB signaling [32]. Additional evidence shows that Lut enhances endothelial resilience through adenosine monophosphate-activated protein kinase activation and protein kinase C regulation, limiting nicotinamide adenine dinucleotide phosphate oxidase assembly and superoxide generation [74] mechanisms that align with the antioxidant and NAD^+^-restoring effects found in our models. Likewise, Lut inhibits TNF-α-derived ROS, and NF-κB-dependent inflammatory signaling [75], collectively representing molecular cascades tightly linked to vascular aging and SASP amplification. More recently, Lut was shown to suppress endothelial ferroptosis through direct activation of the Sirtuin 1/nuclear factor erythroid 2-related factor 2 antioxidant axis, improving cell viability, reducing lipid ROS accumulation, and restoring GPX-dependent redox homeostasis [76]. This is highly consistent with our observation that Lut elevates NAD^+^ and GPX levels and alleviates oxidative damage in aging endothelium. Supporting evidence also comes from studies on other Lut-containing extracts, where Lut and its glycosides block NF-κB activation in human endothelial cells [77], further reinforcing its robust anti-inflammatory and vasculoprotective properties. Collectively, these human-cell-based findings bridge the cross-species results of our study with human endothelial biology, providing strong external validation and enhancing the translational significance of Lut’s anti-vascular-aging mechanisms.

Transcriptomic analysis of SAMP8 aortas revealed that Lut reversed several aging-associated events, including immune activation, lipid accumulation, and mitochondrial dysfunction. Lut upregulated retinol and PPAR signaling, as indicated by the increased expression of *Rdh10*, *Aldh1a1*, *Ppara*, and *Rxrg*. It also promoted cholesterol catabolism and bile acid synthesis via *Cyp7a1* and *Nr1h4* [78,79]. These findings indicate the role of Lut in enhancing lipid clearance and metabolic flexibility. Concurrently, Lut suppressed pro-inflammatory pathways, such as PD-L1, and NF-κB signaling, as evidenced by the reduced expression of *Cd3g*, *Cd274*, *Relb*, and *Tnfaip3*. These changes indicate the occurrence of vascular immunosenescence inhibition and immune–lipid axis rebalancing. This dual modulation mechanism is consistent with earlier findings, in which Lut was found to activate PPARγ while suppressing NF-κB in vascular inflammation models [52,73]. Together, these findings demonstrate that Lut alleviates vascular aging by reprogramming immune and metabolic signaling to a regenerative, anti-inflammatory state.

It should be noted that this study has several limitations. Although our transcriptomic and metabolomic analyses consistently indicated activation of the retinoic acid–PPAR signaling pathway, the causal roles of specific targets within this axis require further validation in future mechanistic studies. In addition, similarly to most pharmacological interventions, the Lut dosage used in our experiments may not directly reflect typical dietary exposure. Therefore, its nutritional relevance and translational applicability warrant further investigation.

Nevertheless, the in vitro and in vivo findings provide insights into vascular aging mechanisms that are likely conserved across species and may help inform future research in human vascular biology. Vascular aging is a major upstream driver of cardiovascular disease, contributing to arterial stiffening, hypertension, endothelial dysfunction, atherosclerosis, and increased stroke risk. Because the retinoic acid–PPAR axis regulates lipid metabolism, vascular inflammation, and mitochondrial homeostasis, the restoration of this pathway by Lut observed in our models suggests potential value for maintaining vascular function during aging. These findings offer a mechanistic basis for considering Lut or Lut-rich dietary patterns as nutritional strategies that may support vascular health and reduce age-related cardiometabolic vulnerability. Future studies evaluating physiological exposure levels, long-term effects, and practical formulations will be essential for advancing Lut as a dietary component with potential relevance to vascular aging and health maintenance.

## 5. Conclusions

In conclusion, this study demonstrates that Lut alleviates vascular aging by restoring retinoic acid–PPAR signaling and promoting lipid metabolic remodeling across both cellular and murine aging models. By integrating senescence phenotyping with transcriptomic and metabolomic analyses, we identified a conserved regulatory network in which Lut enhances retinoid availability, activates PPAR-dependent lipid turnover, and attenuates inflammatory and oxidative pathways associated with endothelial senescence. The convergence of molecular, metabolic, and phenotypic evidence across species highlights a robust and reproducible anti-aging effect on vascular tissues. Collectively, these findings position Lut as a promising dietary component with potential relevance for supporting vascular health during aging.

## Data Availability

The raw RNA-seq data from cVECs have been deposited in the NCBI Sequence Read Archive (SRA) under BioProject accession number PRJNA1314464. The raw RNA-seq data from murine abdominal aortas (SAMP8 cohorts) are available in the NCBI SRA under BioProject accession number PRJNA1314920. The raw metabolomics data from cVECs have been deposited in the Zenodo open access repository developed by CERN and the European Commission (DOI: 10.5281/zenodo.17044381). All other relevant data supporting the findings of this study are provided in the Supplementary Information and within the manuscript.

## Supplementary Materials

The following supporting information can be downloaded at: https://www.mdpi.com/article/10.3390/nu17223607/s1, Supplementary Materials (.doc): The detailed methods for RNA sequencing and untargeted metabolomics. Figure S1. Transcriptomic profiling of cVECs reveals p53 signaling pathway activation by Dox and modulation by Lut. Figure S2. Transcriptomic analysis of abdominal aorta tissues in aged SAMP8 mice after 8-week oral administration of Lut. Supplementary Table (.xlsx): Table S1: Core enriched genes in the p53 signaling pathway (Dox vs. NC) identified by GSEA; Table S2: Core enriched genes in the cAMP signaling pathway (Dox + Lut vs. Dox) identified by GSEA.

## Abbreviations

The following abbreviations are used in this manuscript:

Lut	Luteolin
cVECs	Canine vascular endothelial cells
fVECs	Feline vascular endothelial cells
RA	Retinoic acid
PPAR	Peroxisome proliferator-activated receptor
RXR	Retinoid X receptor
*ALDH1*	Aldehyde dehydrogenase 1
DOX	Doxorubicin
ROS	Reactive oxygen species
SOD	Superoxide dismutase
GPX	Glutathione peroxidase
CAT	Catalase
VEGF	Vascular endothelial growth factor
bFGF	Basic fibroblast growth factor
IL	Interleukin
TNF	Tumor necrosis factor
MMP	Matrix metalloproteinase
MCP	Monocyte chemoattractant protein
SA-β-Gal	Senescence-associated β-galactosidase
pNPG	p-nitrophenyl-β-D-galactopyranoside
SAMP8	Senescence-accelerated mouse prone 8
DEG	Differentially expressed gene
PCA	Principal component analysis
cAMP	Cyclic adenosine monophosphate
GPCR	G-protein coupled receptor
AC	Adenylyl cyclase
PKA	Protein kinase A
PD-L1	Programmed death-ligand 1
NF-κB	Nuclear factor kappa-light-chain-enhancer of activated B cells
PGE2	Prostaglandin E2
AA	Arachidonic acid
S1P	Sphingosine-1-phosphate
