# Supplementary material for: Luteolin Alleviates Vascular Senescence Through Retinoic Acid–Peroxisome Proliferator-Activated Receptor Signaling and Lipid Metabolism Remodeling Combined with Multi-Omics Analysis
Source: Nutrients. 2025 Nov 19;17(22):3607. doi: 10.3390/nu17223607 (PMC12655561; doi:10.3390/nu17223607)
Supplement: Supplementary file 1 [file nutrients-17-03607-s001.zip › Supplementary Materials.pdf]

Supplementary Materials

Luteolin alleviates vascular senescence through retinoic acid-PPAR signaling and lipid metabolism remodeling combined with multi-omics analysis

Huasong Bai[a,1], Hongchen Jin[a,1], Tong Liu[a], Yulong Yin[b], Hengyan Wang[a], Siyu Ruan[c], Yunliang Li[d,*], Zhanzhong Wang[a,*]

[a] Nourse Science Centre for Pet Nutrition, Wuhu 241200, PR China

[b] Institute of Subtropical Agriculture, Chinese Academy of Sciences, Changsha 410125, PR China

[c] College of Tea and Food Science Technology, Jiangsu Vocational College of Agriculture and Forestry, 19 Wenchangdong Road, Jurong, Jiangsu 212400, PR China

[d] School of Food and Biological Engineering, Jiangsu University, 301 Xuefu Road, Zhenjiang 212013, PR China

*Corresponding author: wzz7698@tju.edu.cn (Z.Z.W) and liyunliang@ujs.edu.cn (Y.L.L)

[1] H.S. Bai and H.C. Jin contributed equally to this work

1 RNA sequencing

RNA quality, including concentration and integrity, was evaluated with the Agilent Bioanalyzer system. Polyadenylated mRNA was enriched using Oligo(dT)-conjugated magnetic beads, followed by first-strand cDNA synthesis using M-MuLV reverse transcriptase, and second-strand synthesis using DNA polymerase I. Then, the resulting cDNA was subjected to end repair, poly(A)-tailing, adaptor ligation, and purification with AMPure XP beads. Final libraries were amplified by PCR and sequenced in paired-end mode on the Illumina NovaSeq 6000 platform (Novogene, Beijing, China).

Raw reads were aligned to the corresponding reference genomes using HISAT2 (v2.2.0): *Canis familiaris* (Ensembl 68, CanFam3.1) and *Mus musculus* (Ensembl 92, GRCm38). Reference indices were generated before alignment. FeatureCounts (v2.0)

was applied to calculate gene-level read counts and FPKM values (fragments per kilobase of transcript per million mapped reads), after which principal component analysis (PCA) was performed on the FPKM profiles. Differentially expressed genes were identified using the DESeq2 R package (v1.42.0), and *P*-values (*p*adj) were adjusted using Benjamini-Hochberg correction. Genes with |fold change (FC)| ≥ 1.2 and *p*adj ≤ 0.05 were considered significantly differentially expressed.

KEGG-based gene set enrichment analysis (GSEA) was conducted using the standalone GSEA software (v3.2). Gene ranking was performed using the Signal2Noise metric to assess expression differences across groups. Enrichment scores (ES) were generated by permutation analysis, with normalized enrichment scores (NES) applied to adjust for gene set size variation. False discovery rates (FDR) were estimated using the GSEA algorithm.

2 Untargeted metabolomics of cVECs

Untargeted metabolomic analyses were carried out to investigate treatment-dependent metabolic variations in cVECs. Analyses were performed on an LC-MS/MS platform. To monitor reproducibility, pooled quality control samples were prepared by mixing identical fractions from all extracts, and 53% methanol was included as a solvent blank. The separation of metabolites was performed using an UHPLC system equipped with a Hypersil Gold C18 column, and the eluted compounds were examined on a Q Exactive HF Orbitrap mass spectrometer (Thermo Fisher Scientific, USA) under both positive and negative ionization conditions. Peak detection and quantification were carried out using XCMS. Metabolites were identified using the mzCloud, mzVault, and MassList databases. Relative quantification was performed using Python (v3.5). Background ions were subtracted from blank samples, and compounds with a coefficient of variation >30% in QC samples were excluded. Metabolite intensities

were normalized by dividing sample signal intensities by their corresponding QC values. PCA was conducted using metaX. VIP scores, FC, and T-tests were applied to identify differential metabolites, with significance defined as P <0.05, FC ⩾ 1.5 or ⩽ 0.67, and VIP ⩾ 1. Volcano plots were generated using R (v3.4.3). Functional classification and pathway enrichment were annotated using the KEGG database, and pathway significance was assessed using a hypergeometric test.

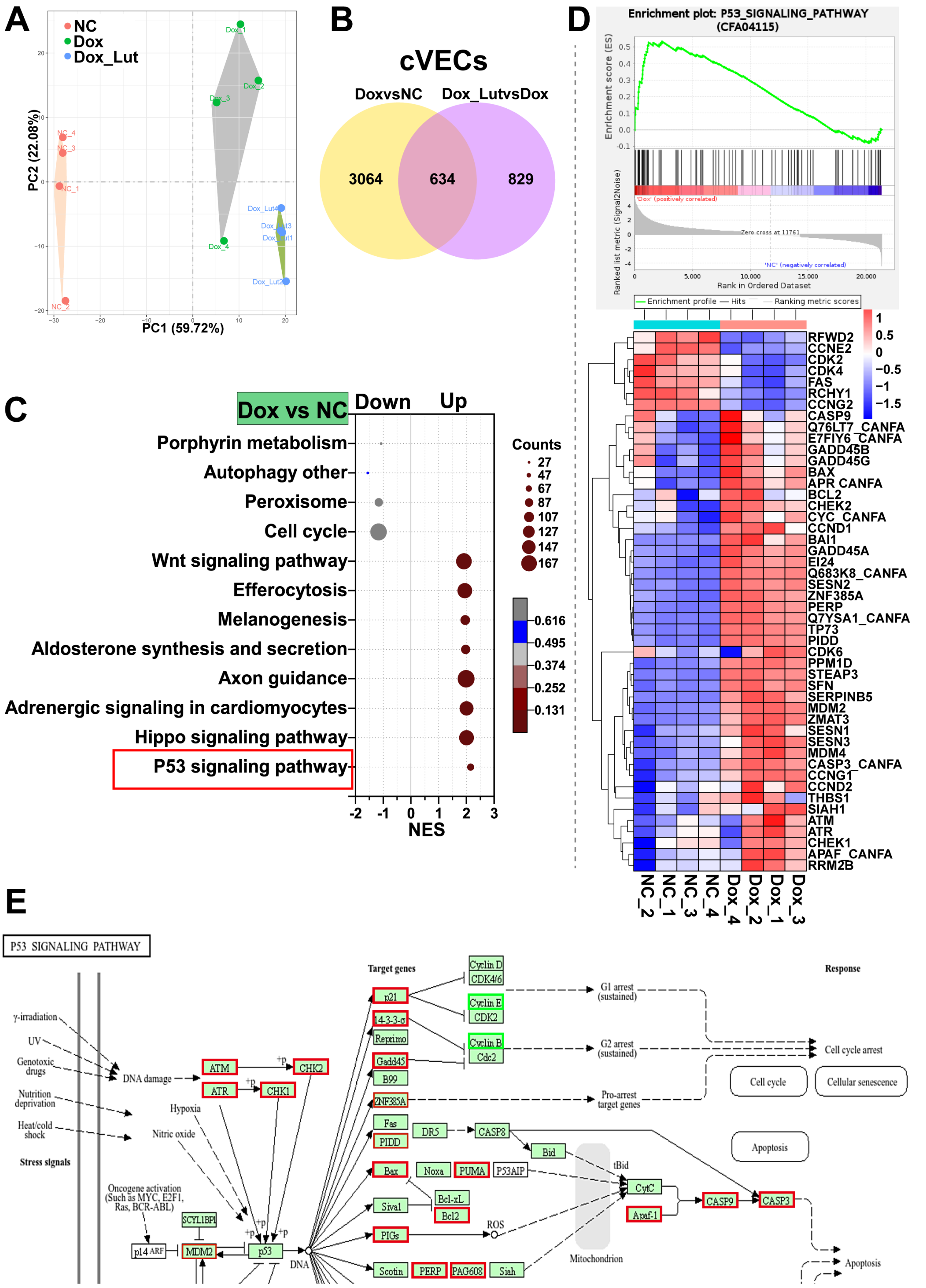

Fig. S1. Transcriptomic profiling of cVECs reveals p53 signaling pathway activation by Dox and modulation by luteolin. **(A)** Principal component analysis (PCA) plot showing distinct transcriptomic profiles of cVECs in NC (vehicle control), Dox (0.8 μM), and Dox + Lut (0.8

μM Dox + 0.625 μM Lut) treatment groups (n = 4). **(B)** Venn diagram showing the number of differentially expressed genes (DEGs) in Dox vs. NC and Dox_Lut vs. Dox comparisons, and their overlap. **(C)** KEGG pathway enrichment of DEGs in Dox vs. NC. The x-axis shows normalized enrichment scores (NES); positive values represent pathway activation, while negative values indicate repression. Dot size reflects gene count; dot color indicates FDR-adjusted significance level (q-value). **(D)** Gene set enrichment analysis (GSEA) of the p53 signaling pathway in Dox vs. NC. Top: running enrichment score (ES) curve for the pathway. Middle: position of p53-related genes in the ranked gene list. Bottom: hierarchical clustering heatmap showing expression of core enrichment genes in each sample. **(E)** KEGG diagram of the p53 signaling pathway. Red-framed genes are significantly upregulated and green-framed genes are downregulated in Dox vs. NC.

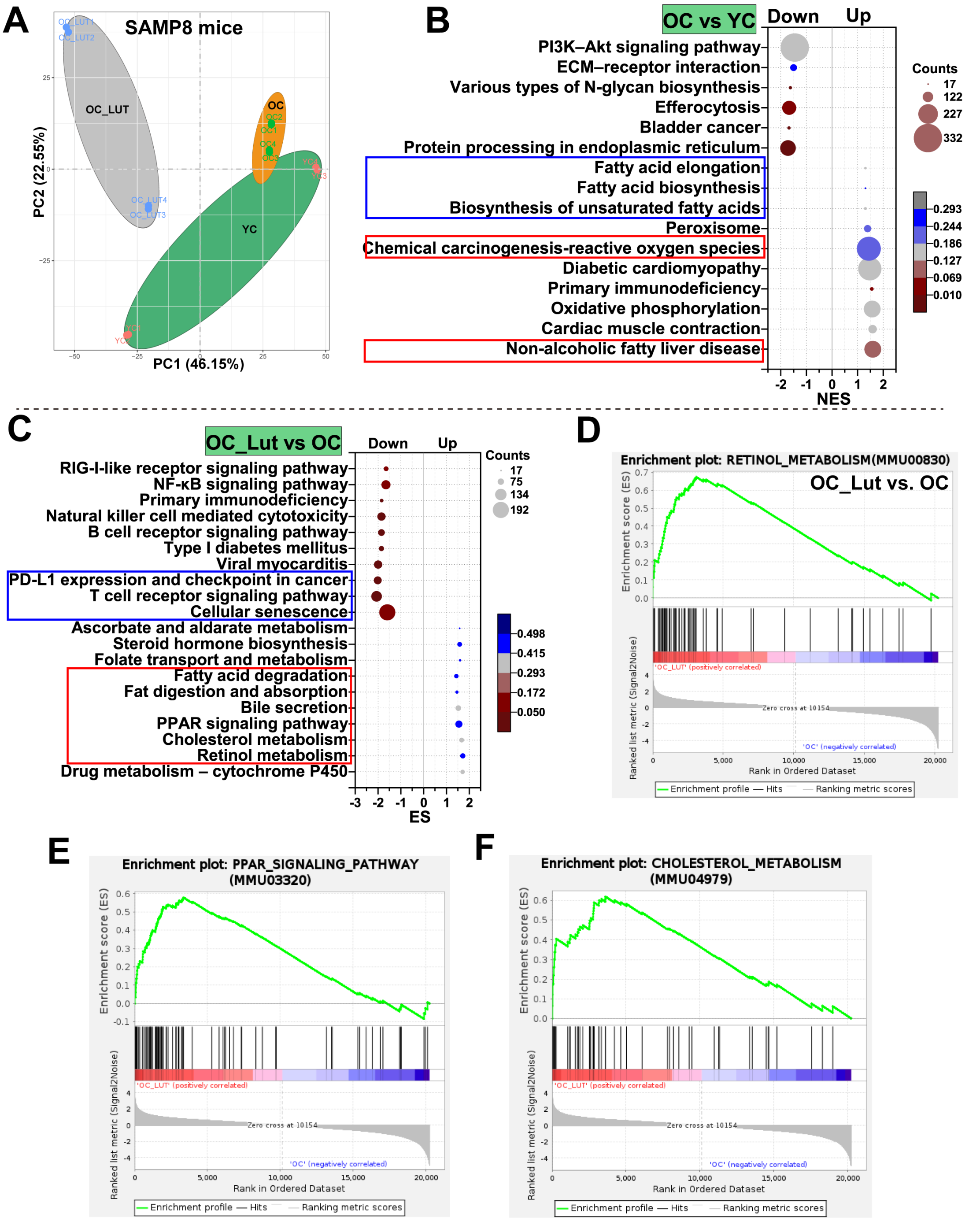

Fig. S2. Transcriptomic analysis of abdominal aorta tissues in aged SAMP8 mice after 8-week oral administration of Lut. (A) Principal component analysis (PCA) of gene expression profiles in young control (YC), aged control (OC), and aged Lut-treated (OC_Lut) mice. (B) KEGG pathway enrichment in OC vs. YC based on GSEA. (C) KEGG pathway enrichment in OC_Lut vs. OC based on GSEA. Upregulated pathways included retinol metabolism, PPAR signaling,

cholesterol metabolism, and fatty acid degradation. (D) Enrichment plots of representative upregulated pathways in OC_Lut vs. OC: retinol metabolism, PPAR signaling pathway, and cholesterol metabolism. The y-axis indicates the running enrichment score (ES), and the peak represents the maximum ES. Gene distribution is shown on the x-axis.
